# Changes in Body Composition in Anorexia Nervosa: Predictors of Recovery and Treatment Outcome

**DOI:** 10.1371/journal.pone.0143012

**Published:** 2015-11-23

**Authors:** Zaida Agüera, Xandra Romero, Jon Arcelus, Isabel Sánchez, Nadine Riesco, Susana Jiménez-Murcia, Jana González-Gómez, Roser Granero, Nuria Custal, Monica Montserrat-Gil de Bernabé, Salomé Tárrega, Rosa M. Baños, Cristina Botella, Rafael de la Torre, José C. Fernández-García, José M. Fernández-Real, Gema Frühbeck, Javier Gómez-Ambrosi, Francisco J. Tinahones, Ana B. Crujeiras, Felipe F. Casanueva, José M. Menchón, Fernando Fernández-Aranda

**Affiliations:** 1 Department of Psychiatry, University Hospital of Bellvitge-IDIBELL, Barcelona, Spain; 2 CIBER Fisiopatología de la Obesidad y la Nutrición (CIBERobn), Instituto Salud Carlos III, Madrid, Spain; 3 Loughborough University Centre for Research into Eating Disorders, Loughborough University, Loughborough, United Kingdom; 4 Department of Clinical Sciences, School of Medicine, University of Barcelona, Barcelona, Spain; 5 Marqués de Valdecilla Public Foundation-Research Institute (FMV-IFIMAV), Santander, Spain; 6 Department of Psychobiology and Methodology, Universitat Autònoma de Barcelona, Barcelona, Spain; 7 Dietetics and Nutrition Unit, University Hospital of Bellvitge, Barcelona, Spain; 8 Department of Personality, Evaluation and Psychological Treatment, University of Valencia, Valencia, Spain; 9 Department of Basic Psychology, Clinic and Psychobiology, University Jaume I, Castelló, Spain; 10 Human Pharmacology and Clinical Neurosciences Research Group, Neuroscience Research Program, IMIM (Hospital del Mar Medical Research Institute), Barcelona, Spain; 11 Department of Diabetes, Endocrinology and Nutrition, Hospital Clínico Universitario Virgen de Victoria, Málaga, Spain; 12 Department of Diabetes, Endocrinology and Nutrition, Institut d’Investigació Biomèdica de Girona (IdlBGi) Hospital Dr Josep Trueta, Girona, Spain; 13 Department of Endocrinology and Nutrition, Clínica Universidad de Navarra, University of Navarra, Pamplona, Spain; 14 Endocrine Division, Complejo Hospitalario U. de Santiago, Santiago de Compostela, Spain; 15 CIBER de Salud Mental (CIBERSAM), Barcelona, Spain; Charité-Universitätsmedizin Berlin, Campus Benjamin Franklin, GERMANY

## Abstract

The restoration of body composition (BC) parameters is considered to be one of the most important goals in the treatment of patients with anorexia nervosa (AN). However, little is known about differences between AN diagnostic subtypes [restricting (AN-R) and binge/purging (AN-BP)] and weekly changes in BC during refeeding treatment. Therefore, the main objectives of our study were twofold: 1) to assess the changes in BC throughout nutritional treatment in an AN sample and 2) to analyze predictors of BC changes during treatment, as well as predictors of treatment outcome. The whole sample comprised 261 participants [118 adult females with AN (70 AN-R vs. 48 AN-BP), and 143 healthy controls]. BC was measured weekly during 15 weeks of day-hospital treatment using bioelectrical impedance analysis (BIA). Assessment measures also included the Eating Disorders Inventory-2, as well as a number of other clinical indices. Overall, the results showed that AN-R and AN-BP patients statistically differed in all BC measures at admission. However, no significant time×group interaction was found for almost all BC parameters. Significant time×group interactions were only found for basal metabolic rate (*p* = .041) and body mass index (BMI) (*p* = .035). Multiple regression models showed that the best predictors of pre-post changes in BC parameters (namely fat-free mass, muscular mass, total body water and BMI) were the baseline values of BC parameters. *Stepwise* predictive logistic regressions showed that only BMI and age were significantly associated with outcome, but not with the percentage of body fat. In conclusion, these data suggest that although AN patients tended to restore all BC parameters during nutritional treatment, only AN-BP patients obtained the same fat mass values as healthy controls. Put succinctly, the best predictors of changes in BC were baseline BC values, which did not, however, seem to influence treatment outcome.

## Introduction

Body composition (BC) is considered an accurate measurement of nutritional status and health risk [1,2]. The main components of BC are fat mass (FM), total body water (TBW) and fat-free mass (FFM) [composed of bone and muscle mass (MM)] [3]. The Food and Agriculture Organization of the United Nations (FAO) have reported that, among the general population of women, 20–30% of the total body weight consists of FM [4], and the TBW in healthy adult women accounts for 45%–60% of total body weight [5].

Anorexia nervosa (AN) is a mental disorder that results in extreme body weight loss and high levels of malnutrition due to food restriction [6]. Because of low weight levels, it is not surprising that patients with AN have been found to exhibit multiple metabolic disturbances and a significant decrease in FM and FFM [7], as well as high levels of TBW [8]. Although both NICE and APA treatment guidelines consider the recovery of body weight and the restoration of BC to be one of the most important goals in the treatment of patients with AN, little research has been carried out in this area. The scarce literature on this topic shows rates of FM recovery ranging from 21% to 78% of the total weight gain [7,9–11]. Research in this field has already described that initial weight increases during the refeeding phase is primarily due to increased FM, as FFM increases occur more slowly over time [7,12]. Moreover, some studies have shown that adult female patients with AN present a disproportionate increase of trunk fat after weight gain, resulting in central adiposity [13–15]. However, it seems that this abnormal fat distribution normalizes with time as long as weight recovery is maintained [16]. Additionally, AN patients increase TBW levels during the initial phases of nutritional therapy and this could lead to edema as a consequence [17]. Patients with AN may also experience higher basal metabolic rate (BMR) during refeeding treatment and usually need to eat more calories than healthy individuals to maintain the same healthy weight [18].

When considering AN subtypes, some differences have been found between binge/purging (AN-BP) and restrictive (AN-R) subtypes. Whereas the former presents a greater percentage of FM, the latter has been found to require greater caloric intake to achieve weight restoration [19]. However, 3 to 6 months after achieving healthy weight, both groups tend to normalize their metabolism to the same level as healthy controls [18]. Despite these BC differences, just two studies have assessed AN-R and AN-BP separately [10,20], and these studies present several shortcomings, such as lack of a control group or information about TBW or BMR.

The present study attempts to overcome some of the limitations of previous studies by including a larger and more homogeneous sample of both AN-R and AN-BP patients who were objectively assessed and monitored weekly throughout treatment and for a one-year follow-up. Furthermore, this is the first study, to our knowledge, that longitudinally investigates the role of BC parameters as predictors of treatment outcome. We expect that the findings of this study could provide useful information in the development of nutritional guidelines and treatments for patients with AN by taking these two disorder subtypes into consideration. Thus, the specific aims of the study were: 1) to assess the impact of diagnostic subtype on the nutritional status (i.e. BC and BMR) of AN patients; 2) to describe the changes of BC distribution and BMR in a large sample of AN patients over time compared to a sample of healthy controls; 3) to analyze clinical and psychological predictors of change in BC during treatment; and 4) to analyze predictors (mainly BC parameters) of treatment outcome and dropout rates.

## Materials and Methods

### Participants

The final sample included 118 adult females with anorexia nervosa (70 AN-R and 48 AN-BP), and 143 normal eating/weight controls without the history of an eating disorder (HC). Eating disorder (ED) diagnosis was originally made according to DSM-IV-TR criteria [21] and determined by experienced psychologists and psychiatrists. These diagnoses were reanalyzed *post hoc* using DSM-5 criteria [6]. The imbalance between the number of AN subtypes is due to the fact that the sample consisted of patients who were consecutively admitted for assessment and treatment at the Eating Disorders Unit (Department of Psychiatry, University Hospital of Bellvitge, Barcelona), between 2008 and 2012.

The AN-R sample had a mean age of 25.43 years (SD = 7.77) and a baseline body mass index (BMI) of 16.71 (SD = 1.03). A majority of the patients were single (80.0%) and had completed high school (41.5%). Approximately 63.5% of the AN-R sample was employed. The AN-BP sample had a mean age of 28.27 years (SD = 8.04) and a baseline BMI of 17.21 (SD = 1.36). 73.9% were single and slightly more than half of them had completed high school (51.2%). 63.4% of the AN-BP sample was employed. The HC sample comprised women who were visiting the hospital for routine blood tests and students who volunteered to take part in the study. The mean age of the HC sample was 25.23 years (SD = 7.11) and had a mean baseline BMI of 20.78 (SD = 1.98). Most of the HC sample was single (73.6%), had completed high school (58.1%) and were employed (49.3%).

From our initial sample of 152 AN patients, the following groups of individuals were excluded from the present analysis: a) patients with incomplete questionnaires (n = 23), b) patients with endocrine disorders such as diabetes mellitus, diabetes insipidus and hypo- or hyperthyroidism (n = 2) and c) males (n = 9) as the number of men with this diagnosis was too small to make meaningful comparisons. The exclusion criteria for the healthy-eating control group were: a) being male, b) being younger than 18, c) presenting a lifetime history of ED and a BMI below 18.5 kg/m^2^ or higher than 25 kg/m^2^ (n = 17).

### Assessment

*- Eating Disorders Inventory-2 (EDI-2*; [22]. This is a reliable and valid 91-item multidimensional self-report questionnaire that assesses cognitive and behavioral characteristics in ED. The EDI-2 retains the eight scales found in the EDI (drive for thinness, bulimia, body dissatisfaction, ineffectiveness, perfectionism, interpersonal distrust, interoceptive awareness, and maturity fears) and adds 27 new items to three scales: asceticism, impulse regulation, and social insecurity. All of these scales are answered on a 6-point Likert scale, and provide standardized subscale scores. It has been validated in a Spanish population [22] with a mean internal consistency of 0.63 (coefficient alpha).

Anthropometrics and body composition analysis

Height was measured by a stadiometer without participants wearing shoes.

- *Tanita BC-420MA*: Body composition was measured by bioelectrical impedance analysis (BIA) (Tanita BC- 420MA, Tanita Corp. Tokyo, Japan). This leg-to-leg body composition analyzer is a simple, accurate, noninvasive and validated method for assessing BC [23] which provides a small alternating voltage of 90 μA (50/60Hz) via electrodes on metal foot plates. Body weight as well as FM, FFM, MM, TBW, BMR and BMI were calculated for each patient. BC estimates are derived from body fluids making use of proprietary equations based on resistance index, weight, height, age and sex. The manufacturers have kept these equations confidential and they have not been reported in the literature. BIA has been validated against dual-energy X-ray absorptiometry (DEXA) measures and other reference methods [23]. The literature indicates that utilizing BIA is not only efficacious for assessing BC parameters in ED, but also it could be advantageous in the treatment of AN patients [24].

### Procedure

All patients were evaluated and diagnosed at the ED Unit at the University Hospital of Bellvitge by experienced psychologists and psychiatrists. Two face-to-face interviews were conducted: the first interview provided information about current ED symptoms, antecedents and other psychopathological data of interest, and the second interview comprised a psychometrical and anthropometric assessment. Additional demographic information including education, occupation, weight history, impulsive behaviors and other relevant clinical variables regarding the eating disorder were obtained by a face-to-face, standardized, structured interview. The same assessment was repeated at the end of the treatment and at a one-year follow-up.

The assessment of BC was obtained weekly, always on the same day of the week and at the same time (between 9:00 a.m. and 9:30 a.m.), using the above-described BIA (Tanita BC-420MA) and according to the principles previously described by Kyle et al. [3].

### Treatment

Every patient with AN received the same day-hospital (DH) treatment that combined nutritional, dietary and group cognitive-behavioral therapy (CBT) treatment for patients with AN. This manualized treatment consisted of cognitive-behavioral techniques including stimulus control therapy, exposure and response prevention, cognitive restructuring, problem solving training, social skills, relaxation techniques and relapse prevention. Moreover, individuals received family psychoeducation in a group setting. Patients attended the DH from 9 a.m. to 3 p.m., five days a week (Monday—Friday), for a period of 15 weeks. Food intake was monitored twice during breakfast and lunch (the main food intake of the day).

As part of the DH treatment, weekly weight monitoring was carried out, along with an assessment of eating behavior and cognitions using self-report measures. Patients were re-evaluated at the end of their treatment and categorized into four categories: “full remission”, “partial remission”, “non-remission” and "dropout" (i.e. voluntary treatment discontinuation). These categories were based on treatment outcomes according to DSM-5 criteria [6].

### Nutritional treatment

The nutritional guidelines for patients in the DH are established by the Dietetics and Nutrition Unit at the University Hospital of Bellvitge. Caloric requirement is determined according to a basic diet of 2300 kcal/day corresponding to a balanced distribution of macro- and micronutrients, 60% carbohydrate, 25–30% fat and 15% protein. All patients received a progressive diet split into four phases (starting at 1200 kcal/day initially, and increasing energy intake to 2300 kcal/day) to avoid refeeding syndrome.

### Ethics Statement

The followed procedures were agreed upon and approved by the Ethics Committee of our institution (namely the Ethics Committee of Clinical Research at the University Hospital of Bellvitge) in accordance with the Helsinki Declaration of 1975 as revised in 1983. We obtained signed informed consent from all participants (all patients were adults; no minors were involved in this study).

### Statistical analysis

Statistical analysis was carried out with SPSS20 for Windows (SPSS Inc). Analysis of variance (ANOVA) procedures compared the mean of clinical variables between diagnostic subtypes (AN-R, AN-BP and HC), defining post-hoc multiple contrasts through Bonferroni estimations to measure pairwise comparisons. Pre-post differences (values taken at the start and at the end of treatment) for BC measures were analyzed through a mixed-design ANOVA, defining time (pre- and post-scores) as the intra-factor and group (AN-R and AN-BP) as the inter-factor. Main effects for the time factor were estimated and interpreted in the case of significant time×group interactions and single effects were estimated-interpreted for non-significant time×group interactions.

Multiple regression models determined the main predictors of pre-post changes in BC measures in the AN sample. The ENTER procedure was defined to simultaneously include the following potential predictors: BC measures at baseline (i.e. the level of each BC parameter at admission), patients’ age, duration of the ED, EDI-2 total score and AN-subtype (0 = restricting, 1 = binge/purging). The global predictive capacity of the models was measured using adjusted-R^2^ coefficient.

Finally, logistic regressions assessed the main predictors of therapy outcomes, dropout and remission. Stepwise procedures were defined to automatically select the best discriminative variables, considering the following potential independent variables: BC measures at baseline, patients’ age, duration of the ED, EDI-2 total score and AN-subtype (0 = restricting, 1 = binge/purging). The global predictive capacity of the final models was measured using Nagelkerke’s-R^2^ coefficient.

In cases of multiple comparisons, the Bonferroni correction method has been largely criticized for being too conservative and no standard alternative procedure has been agreed upon. From a practical-clinical perspective, effect sizes are the relevant objective of the analyses (*p*-values are strongly dependent to sample sizes) and thusly, all the effect sizes for the relationships analyzed in this study have been estimated to correct for the use of multiple group comparisons. For pairwise comparisons (in contrast with the ANOVA procedure), effect sizes were measured through Cohen’s d coefficient [25] (|d|<0.5 was considered poor, |d|>0.5 was considered moderate and |d|>0.8 was considered large), with a 95% confidence interval (95%CI) for mean differences (MD) and odds ratio (OR).

## Results

### Comparison between groups at baseline

Table 1 shows the distribution of the eating behavior and BC measures at baseline, and comparisons for the two diagnosis subtype conditions (AN-R and AN-BP) and HC. AN-BP patients were found to significantly differ from HC participants in all variables, and most of the effect sizes of these differences were in the high range (|*d*|>0.80), with the sole exception of perfectionism scale which was moderate (|*d*|≥0.50). AN-R patients also differed from controls in all measures, except for the EDI-2 bulimia score, and the effect sizes of mean differences were in the moderate to high range.

**Table 1:** Clinical comparisons between anorexia nervosa subtypes and healthy controls.

	Means			ANOVA						
	AN-R	AN-BP	HC	Group	AN-BP vs AN-R		AN-R vs HC		AN-BP vs HC	
	*N* = 70	*N* = 48	*N* = 143	*p*	MD	*| d |*	MD	*| d |*	MD	*| d |*
Age of onset (years-old)	18.27	20.43	---	.169	2.16	0.292				
Duration of the ED (years)	6.56	7.87	---	.372	1.31	0.190				
EDI: Drive for thinness	10.95	15.63	2.50	< .001	4.69*	0.731^†^	8.45*	1.413^†^	13.13*	2.727^†^
EDI: Body dissatisfaction	11.56	14.98	5.10	< .001	3.41*	0.428	6.46*	0.925^†^	9.88*	1.364^†^
EDI: Interocep. awareness	8.47	10.63	1.53	< .001	2.16*	0.380	6.94*	1.441^†^	9.11*	2.286^†^
EDI: Bulimia	1.47	5.10	0.63	< .001	3.62*	1.056^†^	0.84	0.439	4.47*	1.359^†^
EDI: Interpersonal distrust	5.58	5.24	1.83	< .001	-0.34	0.076	3.75*	1.049^†^	3.42*	0.912^†^
EDI: Ineffectiveness	9.20	9.78	1.49	< .001	0.58	0.076	7.71*	1.306^†^	8.29*	1.531^†^
EDI: Maturity fears	7.62	7.59	3.57	< .001	-0.03	0.006	4.05*	0.870^†^	4.01*	0.926^†^
EDI: Perfectionism	6.22	5.76	3.60	< .001	-0.46	0.093	2.62*	0.621^†^	2.16*	0.529^†^
EDI: Impulse regulation	5.49	5.97	1.11	< .001	0.48	0.088	4.38*	0.980^†^	4.86*	1.223^†^
EDI: Asceticism	5.58	8.05	1.89	< .001	2.47*	0.573^†^	3.70*	1.095^†^	6.17*	1.852^†^
EDI: Social insecurity	6.65	6.85	1.86	< .001	0.19	0.041	4.80*	1.230^†^	4.99*	1.358^†^
EDI: Total score	78.80	95.38	25.11	< .001	16.58*	0.398	53.69*	1.564^†^	70.28*	2.303^†^
FM%-baseline	8.42	10.50	15.30	< .001	2.08*	0.390	-6.88*	1.367^†^	-4.80*	0.920^†^
FFM (kg)-baseline	40.64	39.38	42.95	< .001	-1.26*	0.395	-2.31*	0.658^†^	-3.57*	1.009^†^
MM (kg)-baseline	38.57	37.36	40.76	< .001	-1.21*	0.396	-2.20*	0.658^†^	-3.40*	1.011^†^
TBW%-baseline	63.66	61.64	54.77	< .001	-2.03*	0.431	8.89*	2.050^†^	6.86*	1.615^†^
Bone-baseline	2.08	2.01	2.19	< .001	-0.06*	0.409	-0.11*	0.653^†^	-0.18*	0.990^†^
BMR-baseline	1224.9	1184.1	1325.1	< .001	-40.77*	0.438	-100.2*	0.995^†^	-141.0*	1.360^†^
BMI -baseline	16.71	17.21	20.78	< .001	0.50*	0.413	-4.07*	2.581^†^	-3.57*	2.101^†^

MD: mean difference. *d*: Cohen’s-*d* measuring effect size for mean difference.

*Significant mean difference (pairwise comparison, contrast) (.05 level).

^†^Moderate (|*d*|≥0.50) to large (|*d*|≥0.80) effect size.

---Measure not available for the HC group.

AN-R: anorexia nervosa restricting subtype. AN-BP: anorexia nervosa binge/purging subtype. BMI: body mass index. BMR: basal metabolic rate. ED: eating disorder. FFM: fat-free mass. FM: fat-mass. HC: healthy controls. MM: muscular mass. TBW: total body water.

Pairwise comparisons between AN-R versus AN-BP subtypes found significant differences for some EDI-2 scales (drive for thinness, body dissatisfaction, interoceptive awareness, bulimia, asceticism and total score), with AN-BP patients obtaining higher EDI-2 scores. Effect sizes for mean differences were in the moderate to high range for EDI-2 drive for thinness, bulimia and asceticism scores. All BC means were also statistically different when comparing AN-R and AN-BP subtypes. AN-BP patients had significantly higher FM and BMI than AN-R patients, though AN-R patients obtained higher mean values in all the remaining BC measures.

### Changes in body composition during treatment

**Fig 1:**
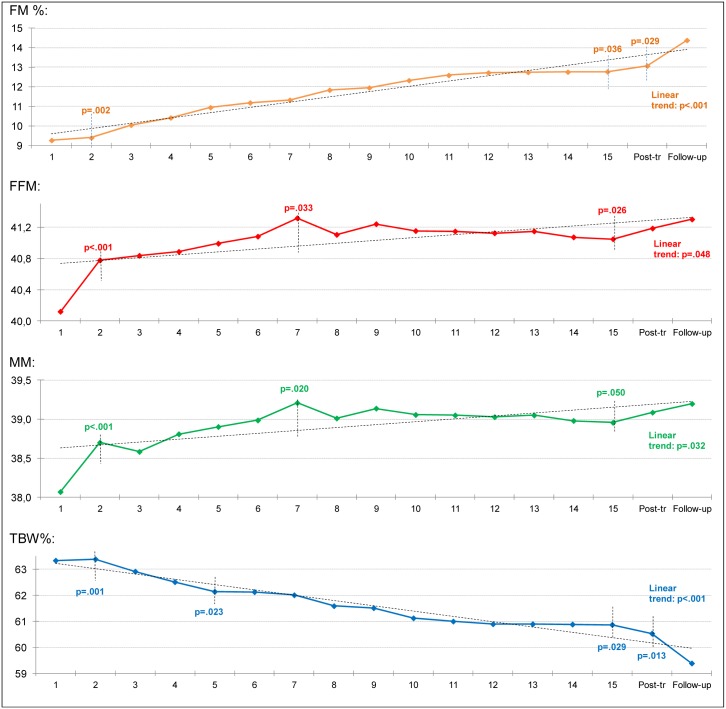
Evolution in body composition in AN patients during treatment. Horizontal dashed-line represents the linear trend and vertical dashed-line indicates a significant change in the slope-trend.

Fig 1 shows the evolution of main BC means for AN patients during therapy and at the 12-month follow-up. The incorporation of polynomial contrasts into the ANOVA procedures showed a significant linear trend for all these measures, indicating an increase in FM, FFM and MM, and a decrease in TBW. The presence of other significant trends during the process showed that changes were not statistically constant during therapy and follow-up. As seen in Fig 1, FM increased regularly and progressively during every week of treatment, whereas FFM and MM showed a sharp increase during the first 7 weeks of treatment, but stabilized at mid-treatment.

Table 2 shows the means for the BC measures at baseline and at the end of treatment, and the comparison of the pre-post mean changes. No significant time×group interaction was obtained for FM, FFM, MM, TBW and bone mass, indicating that mean changes were statistically equal for both diagnostic conditions (AN-R and AN-BP). A significant mean increase was observed for this set of variables at the end of therapy, with the exception of total body water, which registered a significant mean decrease. As per BMR and BMI, a time×group significance was obtained, indicating that pre-post mean changes were different for the two diagnostic conditions: this pre-post mean increase was higher for binge/purging subtype compared to restricting subtype. Effect sizes for the mean changes shown in Table 2 were only in the moderate to high range for FM, TBW, BMR and BMI.

**Table 2:** Changes in body composition for AN patients.

	Mean				Inter.	Factor: Time (difference pre-post)				
	AN-R (*N* = 70)		AN-BP (*N* = 48)		time×gr.	Sign.	Mean	95%CI		Cohen’s
	Pre	Post	Pre	Post	*p*	*p*	differ.	Mean difference		*| d |*
FM%	8.22	11.86	10.15	15.03	.119	< .001	4.26*	3.47;	5.04	0.689^†^
FFM (kg)	40.68	41.42	39.39	40.82	.081	< .001	1.09*	0.70;	1.47	0.344
MM (kg)	38.60	39.31	37.37	38.74	.076	< .001	1.04*	0.67;	1.41	0.345
TBW%	63.51	60.74	61.71	58.30	.259	< .001	-3.09*	-3.65;	-2.53	0.598^†^
Bone	2.08	2.11	2.01	2.08	.178	< .001	0.05*	0.03;	0.07	0.336
BMR	1224.5	1249.8	1182.6	1229.6	.041	< .001^R^	25.30* ^R^	12.24^R^;	38.36^R^	0.273
						< .001^P^	47.02* ^P^	30.84^P^;	63.21^P^	0.506^†^
BMI	16.71	17.69	17.21	18.62	.035	< .001^R^	0.98* ^R^	0.72^R^;	1.23^R^	0.806^†^
						< .001^P^	1.41* ^P^	1.10^P^;	1.72^P^	0.973^†^

*Significant mean difference (pairwise comparison, contrast) (.05 level).

^†^Moderate (|*d*|≥0.50) to large (|*d*|≥0.80) effect size.

^R^Pre-post (at the start and at the end of treatment) comparison for AN- restricting.

^P^Pre-post comparison for AN-binge/purging.

AN-R: anorexia nervosa restricting subtype. AN-BP: anorexia nervosa binge/purging subtype. BMI: body mass index. BMR: basal metabolic rate. FFM: fat-free mass. FM: fat mass. MM: muscular mass. TBW: total body water.

Table 3 shows the means for BC measures at the end of treatment for the AN groups and the means for the HC group. Pairwise comparison between AN-R and HC showed significant differences in all BC measures (effect sizes were moderate to high for FM, TBW, BMR and BMI), with the AN-R sample presenting lower FM, BMR, BMI and higher TBW than the HC group. Comparing AN-BP to HC conditions, all BC means significantly differed (with the exception of FM): AN-BP patients, at the end of the treatment, still had lower FFM, MM, bone mass, BMR and BMI values and higher TBW than the HC sample (effect sizes for significant mean differences were moderate to high). Post-hoc comparisons for AN-BP and AN-R only obtained significant results for FM (higher for binge/purging subtype), total body water (higher for restricting subtype) and BMI (higher for binge/purging patients). Effect sizes for these three statistical differences were in the moderate range.

**Table 3:** Comparisons between body composition at the end of treatment in AN patients and controls.

	Mean			ANOVA						
	AN-R	AN-BP	HC	Group	AN-R vs AN-BP		AN-R vs HC		AN-BP vs HC	
	*N* = 70	*N* = 48	*N* = 143	*p*	MD	*| d |*	MD	*| d |*	MD	*| d |*
FM%	11.86	15.03	15.30	< .001	3.18*	0.583^†^	3.45*	0.661^†^	0.27	0.052
FFM (kg)	41.42	40.82	42.95	< .001	-0.60	0.209	1.53*	0.435	2.13*	0.659^†^
MM (kg)	39.31	38.74	40.76	< .001	-0.56	0.206	1.46*	0.436	2.02*	0.657^†^
TBW%	60.74	58.30	54.77	< .001	-2.52*	0.590^†^	-6.25*	1.434^†^	-3.73*	0.999^†^
Bone	2.11	2.08	2.19	< .001	-0.04	0.257	0.07*	0.420	0.11*	0.689^†^
BMR	1249.8	1229.6	1325.0	< .001	-20.37	0.230	75.10*	0.731^†^	95.46*	0.977^†^
BMI	17.69	18.62	20.78	< .001	0.93*	0.643^†^	3.09*	1.819^†^	2.16*	1.224^†^

MD: mean difference. *d*: Cohen’s-*d* measuring effect size for mean difference.

*Significant mean difference (pairwise comparison, contrast) (.05 level).

^†^Moderate (|*d*|≥0.50) to large (|*d*|≥0.80) effect size.

AN-R: Anorexia nervosa restricting. AN-BP: Anorexia nervosa binge/purging. BMI: body mass index. BMR: basal metabolic rate. FFM: fat-free mass. FM: fat mass. HC: healthy controls. MM: muscular mass. TBW: total body water.

S1 Fig (see “Supporting Information”) shows the evolution of BC means for AN patients at pre-treatment, post-treatment and one-year follow-up, and the comparison with the HC group.

### Predictors of changes in body composition

Table 4 shows the results of multiple regression models assessing what the best predictors of pre-post changes (from the start to the end of therapy) in BC were. These models included BC measures at baseline, for example the model for FM change included the FM value at the moment of admission. No significant predictors emerged for FM and BMR. For FFM, MM, TBW and BMI, the only significant predictors of pre-post changes were these same BC measures at baseline.

**Table 4:** Predictors of pre-post changes in body composition for AN patients.

	**Fat mass (adjusted R^2^ = .051)**						**Fat-free mass (adjusted R^2^ = .037)**					
	**B**	**SE**	***Beta***	***p***	***95%CI(B)***		**B**	**SE**	***Beta***	***p***	***95%CI(B)***	
Intercept	7.706	1.973	---	< .001	3.755	11.657	5.367	2.423	---	.030	0.534	10.201
BC-baseline	-.053	0.080	-.086	.512	-0.213	0.107	**-.114**	**0.055**	**-.256**	**.042**	**-0.224**	**-0.004**
Age (years-old)	-.090	0.064	-.226	.165	-0.218	0.038	.026	0.024	.159	.281	-0.022	0.075
Duration ED (years)	-.068	0.074	-.145	.361	-0.216	0.080	-.032	0.026	-.174	.220	-0.083	0.019
EDI-total score	-.006	0.010	-.076	.579	-0.026	0.014	.005	0.004	.178	.161	-0.002	0.013
AN-subtype (purging)	1.20	0.930	.182	.204	-0.668	3.057	-.098	0.341	-.036	.775	-0.777	0.582
	**Muscular mass (adjusted R^2^ = .035)**						**Total body water (adjusted R^2^ = .028)**					
	**B**	**SE**	***Beta***	***p***	***95%CI(B)***		**B**	**SE**	***Beta***	***p***	***95%CI(B)***	
Intercept	5.060	2.299	---	.031	0.474	9.646	-13.03	3.774	---	.001	-20.56	-5.500
BC-baseline	**-.113**	**0.055**	**-.253**	**.045**	**-0.222**	**-0.003**	**.255**	**0.055**	**.507**	**< .001**	**0.146**	**0.364**
Age (years-old)	.025	0.023	.158	.284	-0.021	0.071	.006	0.041	.019	.884	-0.075	0.087
Duration ED (years)	-.030	0.024	-.174	.221	-0.079	0.019	-.007	0.043	-.022	.865	-0.093	0.079
EDI-total score	.005	0.004	.170	.180	-0.002	0.012	-.002	0.006	-.041	.720	-0.015	0.010
AN-subtype (purging)	-.085	0.324	-.033	.794	-0.731	0.562	.672	0.562	.135	.236	-0.449	1.793
	**Basal metabolic rate (adjusted R^2^ = .046)**						**Body mass index (adjusted R^2^ = .036)**					
	**B**	**SE**	***Beta***	***p***	***95%CI(B)***		**B**	**SE**	***Beta***	***p***	***95%CI(B)***	
Intercept	177.6	83.37	---	.037	11.2	343.9	3.840	1.467	---	.011	0.920	6.760
BC-baseline	-.119	0.060	-.274	.052	-0.239	0.001	**-.179**	**0.083**	**-.241**	**.034**	**-0.344**	**-0.014**
Age (years-old)	.615	0.770	.125	.427	-0.921	2.151	.019	0.016	.159	.227	-0.012	0.050
Duration ED (years)	-1.127	0.751	-.210	.138	-2.624	0.371	-.020	0.017	-.151	.238	-0.054	0.014
EDI-total score	.065	0.113	.072	.565	-0.160	0.290	.000	0.002	.021	.857	-0.004	0.005
AN-subtype (purging)	1.16	9.948	.015	.907	-18.681	21.008	.007	0.213	.004	.976	-0.417	0.430

Multiple regression models (ENTER procedure). Bold: significant parameter. BC measures: measure of each body composition parameter at admission. ED: eating disorder.

### Body composition as a predictor of treatment outcome and dropout rates

No statistical differences due to AN subtype were found for the risk of dropout, total, partial or non-remission in the AN group (χ^2^ = 4.595, df = 3, *p* = .204). Most of the AN-R sample presented full (40%) or partial remission (15.7%), while 20% showed non-remission. Similarly, in the AN-BP sample, 43.8% presented full remission, 27.1% partial remission and 8.3% non-remission. Dropout rates were similar between AN-R (24.3%) and AN-BP patients (20.8%).

Table 5 shows the *stepwise* predictive logistic regressions for therapy outcome. This model contains the best predictors (statistically significant independent variables) of dropout and remission (full or partial+full) during therapy, while also considering the patients’ baseline state. No BC parameter emerged as a predictor of treatment outcome. The best predictor of dropout during therapy was age, (the younger the AN patient, the higher the risk of dropout). BMI was the best discriminative variable of "full remission" outcome (the higher BMI at baseline, the higher the probability of full remission). The probability of partial or full remission was higher for patients who were older and had high BMI.

**Table 5:** Significant predictors of treatment outcome for AN patients.

Criterion (outcome)	Predictors	B	SE	Wald_(1)_	*p*	OR	95%CI (OR)	
Dropout	Intercept	0.676	0.853	0.628	0.428	1.966	---	---
*(Nag-R* ^*2*^ *=* .*071)*	Age (years-old)	-0.075	0.034	4.814	.028	0.928	0.868	0.992
Full remission	Intercept	-9.005	3.121	8.322	.004	.000	---	---
*(Nag-R* ^*2*^ *=* .*098)*	BMI at baseline	0.510	0.183	7.802	.005	1.665	1.164	2.381
Partial or Full remission	Intercept	-11.056	3.275	11.394	.001	.000	---	---
*(Nag-R* ^*2*^ *=* .*171)*	Age (years-old)	0.064	0.028	5.437	.020	1.068	1.010	1.126
	BMI at baseline	0.585	0.181	10.429	.001	1.796	1.259	2.562

Logistic regression models (STEPWISE procedure). Nag-R^2^: Nagelkerke’s-pseudo-R^2^. BMI: body mass index.

## Discussion

The first main finding of the present study was that both AN-R and AN-BP patients showed altered BC compared to healthy controls, which is consistent with the findings of other authors [7,10,11,14]. Furthermore, AN-R and AN-BP patients showed differences in all the BC parameters measured. In line with a prior study [20], AN-R patients had a lower percentage of FM than AN-BP, but a greater percentage of FFM, MM and TBW. This could be explained by the high levels of physical activity found among those with AN-R [26]. Patients with compulsive exercising have a lower frequency of binge eating, vomiting, and laxative abuse than non-exercisers [27], and the fact that AN-BP patients have more prevalence of binge eating episodes may also explain why these patients had more FM than restrictive ones.

Also, AN-R patients showed higher BMR than AN-BP. This is in line with other authors [19] reporting that AN-R requires higher caloric intake than AN-BP to maintain a stable weight. Accordingly, a previous study revealed that a history of bulimic symptoms in AN patients may lead to lower caloric requirements [28]. Thus, these data highlight the differences in energy-metabolism efficiency between diagnostic subtypes.

### Body composition changes and weight recovery in AN

Our hypothesis that BC in AN patients would be normalized after the refeeding treatment was partially supported by the present findings. Changes in all BC parameters were observed throughout the nutritional therapy, but only patients with AN-BP reached the same percentage of FM as the control group after treatment, which may be an integral part of recovery. However, all the BC parameters obtained after therapy were close to published 50 percentile values in healthy women [4]. These changes were large for FM, FFM and TBW, and were maintained after one-year follow-up. Otherwise, all the differences between AN-BP and AN-R patients at admission were non-existent at the end of treatment, with the exceptions of FM, BMI and TBW. Furthermore, we observed an interaction between the magnitude of the change in BMR and diagnostic subtype, with BMR increases in AN-BP patients being greater than in AN-R patients. This may be due to AN-BP patients presenting lower BMR at admission than restricting patients. Contrary to a previous study [18], our results did not show that AN patients became hypermetabolic during re-nutrition treatment. Similarly, Bossu et al [29] found that constitutionally lean women (without AN) presented lower BMR than normal-weight women, suggesting an adaptive role for BMR.

An intriguing finding was that FM constantly and progressively increased during every week of treatment, while FFM and the MM showed a rapid increase during the first 7 weeks of treatment, but stabilized at mid-treatment. These results are consistent with a prior study indicating that, in patients with a very low BMI and an initial FM of 4 kg, FFM initially increases more than FM [30]. However, our results are not consistent with other authors [7,12] indicating a greater initial increase of FM and only a progressive, minor gain of FFM during the same period of treatment. These discrepancies might be due to differences in the malnourished state of patients at admission, as proposed Yamashita et al. [30]. Regarding body water, our results failed to find an increase in TBW during the first weeks of refeeding, as Krahn et al [17] reported. In contrast, our results showed that following the second week of nutritional therapy, TBW levels decreased progressively. Overall, the present study has the strength that it is the first to consecutively measure the BC of a large sample of adult AN patients on a weekly basis, unlike in other studies in which BC assessment is performed, at most, at four points during the whole course of treatment.

### Body composition as a predictor of changes in psychopathology

In terms of BC improvement (measured through pre–post differences), we found that the baseline values of BC parameters were the best predictors of change for most of the anthropometric parameters. In this sense, presenting lower levels of FFM, MM and BMI and higher TBW at baseline were associated with greater positive changes in these parameters. However, contrary to previous studies [2,31], our results did not find associations between clinical variables, such as age or duration of the disorder, and changes in BC. These discrepancies could be due to the fact that most differences were found comparing adolescents to adults, but in our case, all the participants were adults with AN. Finally, our findings were not able to uncover a positive association between eating disorder psychopathology and changes in BC. These results are in agreement with those published by El Ghoch et al. [15], who suggested that changes in BC were not associated with distress, body image or eating disorder psychopathology.

### Body composition as a predictor of treatment outcome and dropout rates

Treatment outcome for AN-R patients was similar overall to AN-BP patients. In terms of predictors, our findings suggest that higher BMI as well as older age were positively associated with better treatment outcomes. Accordingly, a low BMI at the beginning of treatment has been identified as one of the most important risk factors for poor prognosis in AN [32]. However, contrary to our hypothesis, our findings were not able to uncover a direct association between low FM and poor clinical outcome in AN patients [18]. One possible explanation may be that low FM is a predictor of poor prognosis only when the patients are very severe and present extremely low values of FM. In our sample, patients were in DH treatment and therefore, they were less severely affected by the disorder and less resistant to treatment than individuals receiving in-patient treatment.

The present study should be evaluated within the context of several limitations. First, we only included adult female AN patients from Spain. Hence, we do not know whether our results are generalizable to adolescent ED patients, males or individuals from other ethnic backgrounds. Second, the BC analyzer we used does not enable the measurement of regional BC and thus does not allow us to assess whether our sample presented central adiposity after refeeding treatment. Finally, the present study failed to consider hormones strongly related to metabolism (mainly leptin) and FM. Since a positive correlation between leptin levels and FM has been well-documented [33], and an association between a lower presence of leptin in AN patients with disease duration has been found [34], finding hormonal changes, such as an increase in leptin being associated with FM recovery, could be expected. It would be of interest to study these associations in order to elucidate the specific role of these hormones in the timing of body composition restoration. Therefore, future studies should aim to collect this information and to replicate this study while assessing hormones and using a segmental BC analyzer to assess adiposity distribution.

In spite of these limitations, the current study also features several strengths. This study has addressed, for the first time, the BC of both AN-R and AN-BP patients using weekly monitoring and a one-year follow-up. The second strength is the availability of a large sample of healthy controls for comparison. Furthermore, this is the first study assessing BC predictors of outcome, and analyzing predictors of change in BC compartments during refeeding treatment, which, to our knowledge, has not been attempted before.

The findings from the present study may help to shape appropriate nutritional guidelines and treatment programs for patients with AN. As the review by Saladino [24] suggests, the assessment of BC changes during the treatment of AN patients might allow for the development of individualized nutritional diets for each patient. Even though our results showed differences between AN-R and AN-BP in BC distribution at admission, it seems that both AN-R and AN-BP patients would benefit equally from the same nutritional treatment. Given the high mortality rate found in AN [35], it is imperative to study BC to develop nutritional guidelines and cost-effective methods of treatment.

## Conclusions

In conclusion, our findings confirm the progressive improvement in all BC parameters in adult AN patients during DH nutritional treatment. Both AN-R and AN-BP patients showed differences in BC distribution at admission, but these differences do not appear to influence BC recovery, with the exception of BMR. It is also noteworthy that the best predictors of BC changes were baseline BC values. Curiously, no BC parameter was found to be a predictor of outcome, only higher baseline BMI and older age were associated with better treatment outcomes and lower risk of dropout.

## Supporting Information

S1 Fig Body composition in AN compared with healthy controls. (TIF)

## References

[1] Gómez-Ambrosi J, Silva C, Galofré JC, Escalada J, Santos S, Millán D, et al Body mass index classification misses subjects with increased cardiometabolic risk factors related to elevated adiposity. Int J Obes (Lond). 2012;36: 286–94. 10.1038/ijo.2011.100 21587201

[2] De Alvaro MTG, Muñoz-Calvo MT, Barrios V, Martínez G, Martos-Moreno GA, Hawkins F, et al Regional fat distribution in adolescents with anorexia nervosa: effect of duration of malnutrition and weight recovery. Eur J Endocrinol. 2007;157: 473–9. 10.1530/EJE-07-0459 17893262

[3] Kyle UG, Bosaeus I, De Lorenzo AD, Deurenberg P, Elia M, Gómez JM, et al Bioelectrical impedance analysis—part I: review of principles and methods. Clin Nutr. 2004;23: 1226–43. 15380917 10.1016/j.clnu.2004.06.004

[4] Kyle UG, Genton L, Slosman DO, Pichard C. Fat-free and fat mass percentiles in 5225 healthy subjects aged 15 to 98 years. Nutrition. 2001;17: 534–41. Available: http://www.ncbi.nlm.nih.gov/pubmed/11448570. 11448570 10.1016/s0899-9007(01)00555-x

[5] Martinoli R, Mohamed EI, Maiolo C, Cianci R, Denoth F, Salvadori S, et al Total body water estimation using bioelectrical impedance: a meta-analysis of the data available in the literature. Acta Diabetol. 2003;40 Suppl 1: S203–6. 10.1007/s00592-003-0066-2 14618473

[6] APA. Diagnostic and statistical manual of mental disorders: DSM-5. Washington, DC: American Psychiatric Association; 2013.

[7] Polito A, Cuzzolaro M, Raguzzini A, Censi L, Ferro-Luzzi A. Body composition changes in anorexia nervosa. Eur J Clin Nutr. 1998;52: 655–62. Available: http://www.ncbi.nlm.nih.gov/pubmed/9756122. 9756122 10.1038/sj.ejcn.1600618

[8] Dempsey DT, Crosby LO, Lusk E, Oberlander JL, Pertschuk MJ, Mullen JL. Total body water and total body potassium in anorexia nervosa. Am J Clin Nutr. 1984;40: 260–9. Available: http://www.ncbi.nlm.nih.gov/pubmed/6465060. 6465060 10.1093/ajcn/40.2.260

[9] Orphanidou CI, McCargar LJ, Birmingham CL, Belzberg AS. Changes in body composition and fat distribution after short-term weight gain in patients with anorexia nervosa. Am J Clin Nutr. 1997;65: 1034–41. Available: http://www.ncbi.nlm.nih.gov/pubmed/9094890. 9094890 10.1093/ajcn/65.4.1034

[10] Probst M, Goris M, Vandereycken W, Van Coppenolle H. Body composition of anorexia nervosa patients assessed by underwater weighing and skinfold-thickness measurements before and after weight gain. Am J Clin Nutr. 2001;73: 190–7. Available: http://www.ncbi.nlm.nih.gov/pubmed/11157313. 11157313 10.1093/ajcn/73.2.190

[11] Scalfi L, Polito A, Bianchi L, Marra M, Caldara A, Nicolai E, et al Body composition changes in patients with anorexia nervosa after complete weight recovery. Eur J Clin Nutr. 2002;56: 15–20. 10.1038/sj.ejcn.1601290 11840175

[12] Mika C, Herpertz-Dahlmann B, Heer M, Holtkamp K. Improvement of nutritional status as assessed by multifrequency BIA during 15 weeks of refeeding in adolescent girls with anorexia nervosa. J Nutr. 2004;134: 3026–30. Available: http://www.ncbi.nlm.nih.gov/pubmed/15514270. 15514270 10.1093/jn/134.11.3026

[13] Mayer L, Walsh BT, Pierson RN, Heymsfield SB, Gallagher D, Wang J, et al Body fat redistribution after weight gain in women with anorexia nervosa. Am J Clin Nutr. 2005;81: 1286–91. Available: http://www.ncbi.nlm.nih.gov/pubmed/15941877. 15941877 10.1093/ajcn/81.6.1286

[14] Iketani T, Kiriike N, Nagata T, Yamagami S. Altered body fat distribution after recovery of weight in patients with anorexia nervosa. Int J Eat Disord. 1999;26: 275–82. Available: http://www.ncbi.nlm.nih.gov/pubmed/10441242. 10441242 10.1002/(sici)1098-108x(199911)26:3<275::aid-eat4>3.0.co;2-i

[15] El Ghoch M, Milanese C, Calugi S, Pellegrini M, Battistini NC, Dalle Grave R. Body composition, eating disorder psychopathology, and psychological distress in anorexia nervosa: a longitudinal study. Am J Clin Nutr. 2014;99: 771–8. 10.3945/ajcn.113.078816 24500157

[16] El Ghoch M, Calugi S, Lamburghini S, Dalle Grave R. Anorexia nervosa and body fat distribution: a systematic review. Nutrients. 2014;6: 3895–912. 10.3390/nu6093895 25251296 PMC4179194

[17] Krahn DD, Rock C, Dechert RE, Nairn KK, Hasse SA. Changes in resting energy expenditure and body composition in anorexia nervosa patients during refeeding. J Am Diet Assoc. 1993;93: 434–8. Available: http://www.ncbi.nlm.nih.gov/pubmed/8454812. 8454812 10.1016/0002-8223(93)92291-5

[18] Marzola E, Nasser JA, Hashim SA, Shih P-AB, Kaye WH. Nutritional rehabilitation in anorexia nervosa: review of the literature and implications for treatment. BMC Psychiatry. 2013;13: 290 10.1186/1471-244X-13-290 24200367 PMC3829207

[19] Kaye WH, Gwirtsman HE, Obarzanek E, George T, Jimerson DC, Ebert MH. Caloric intake necessary for weight maintenance in anorexia nervosa: nonbulimics require greater caloric intake than bulimics. Am J Clin Nutr. 1986;44: 435–43. Available: http://www.ncbi.nlm.nih.gov/pubmed/3766430. 3766430 10.1093/ajcn/44.4.435

[20] Probst M, Goris M, Vandereycken W, Van Coppenolle H. Body composition in female anorexia nervosa patients. Br J Nutr. 1996;76: 639–47. 8957999 10.1079/bjn19960072

[21] APA. Diagnostic and Statistical Manual of Mental Disorders, 4th Edition, Text Revision. 4th ed Washington, DC: American Psychiatric Association; 2000.

[22] Garner DM. Inventario de Trastornos de la Conducta Alimentaria (EDI-2)-Manual. Madrid: TEA; 1998.

[23] Browning LM, Dixon AK, Aitken W, Prentice AM, Jebb A. Measuring Abdominal Adipose Tissue : Comparison of Simpler Methods with MRI. Obes Facts. 2011;4: 9–15. 10.1159/000324546 21372606 PMC6450044

[24] Saladino CF. The efficacy of Bioelectrical Impedance Analysis (BIA) in monitoring body composition changes during treatment of restrictive eating disorder patients. J Eat Disord. 2014;2: 34 10.1186/s40337-014-0034-y 25485109 PMC4258054

[25] Cohen J. Statistical power analysis for the behavioral sciences. (2nd ed.). Hillsdale, NJ: Lawrence Earlbaum Associates; 1988.

[26] Dalle Grave R, Calugi S, Marchesini G. Compulsive exercise to control shape or weight in eating disorders: prevalence, associated features, and treatment outcome. Compr Psychiatry. 2008;49: 346–52. 10.1016/j.comppsych.2007.12.007 18555054

[27] Brewerton TD, Stellefson EJ, Hibbs N, Hodges EL, Cochrane CE. Comparison of eating disorder patients with and without compulsive exercising. Int J Eat Disord. 1995;17: 413–6. Available: http://www.ncbi.nlm.nih.gov/pubmed/7620482. 7620482 10.1002/1098-108x(199505)17:4<413::aid-eat2260170414>3.0.co;2-0

[28] Salisbury JJ, Levine AS, Crow SJ, Mitchell JE. Refeeding, metabolic rate, and weight gain in anorexia nervosa: a review. Int J Eat Disord. 1995;17: 337–45. Available: http://www.ncbi.nlm.nih.gov/pubmed/7620473. 7620473 10.1002/1098-108x(199505)17:4<337::aid-eat2260170405>3.0.co;2-q

[29] Bossu C, Galusca B, Normand S, Germain N, Collet P, Frere D, et al Energy expenditure adjusted for body composition differentiates constitutional thinness from both normal subjects and anorexia nervosa. Am J Physiol Endocrinol Metab. 2007;292: E132–7. 16912058 10.1152/ajpendo.00241.2006

[30] Yamashita S, Kawai K, Yamanaka T, Inoo T, Yokoyama H, Morita C, et al BMI, body composition, and the energy requirement for body weight gain in patients with anorexia nervosa. Int J Eat Disord. 2010;43: 365–71. 10.1002/eat.20700 19459214

[31] Misra M, Soyka LA, Miller KK, Grinspoon S, Levitsky LL, Klibanski A. Regional body composition in adolescents with anorexia nervosa and changes with weight recovery. Am J Clin Nutr. 2003;77: 1361–7. Available: http://www.ncbi.nlm.nih.gov/pubmed/12791610. 12791610 10.1093/ajcn/77.6.1361

[32] Löwe B, Zipfel S, Buchholz C, Dupont Y, Reas DL, Herzog W. Long-term outcome of anorexia nervosa in a prospective 21-year follow-up study. Psychol Med. 2001;31: 881–90. Available: http://www.ncbi.nlm.nih.gov/pubmed/11459385. 11459385 10.1017/s003329170100407x

[33] Bruni V, Dei M, Morelli C, Schettino MT, Balzi D, Nuvolone D. Body Composition Variables and Leptin Levels in Functional Hypothalamic Amenorrhea and Amenorrhea Related to Eating Disorders. J Pediatr Adolesc Gynecol. Elsevier Inc.; 2011;24: 347–352. 10.1016/j.jpag.2011.06.004 21906977

[34] Terra X, Auguet T, Agüera Z, Quesada IM, Orellana-Gavaldà JM, Aguilar C, et al Adipocytokine levels in women with anorexia nervosa. Relationship with weight restoration and disease duration. Int J Eat Disord. 2013;46: 855–61. 10.1002/eat.22166 23881663

[35] Arcelus J, Mitchell AJ, Wales J, Nielsen S. Mortality rates in patients with anorexia nervosa and other eating disorders. A meta-analysis of 36 studies. Arch Gen Psychiatry. 2011;68: 724–31. 10.1001/archgenpsychiatry.2011.74 21727255

