# Supplementary figures and images for: Changes in Body Composition in Anorexia Nervosa: Predictors of Recovery and Treatment Outcome
Source: PLoS One. 2015 Nov 23;10(11):e0143012. doi: 10.1371/journal.pone.0143012 (PMC4658117; doi:10.1371/journal.pone.0143012)

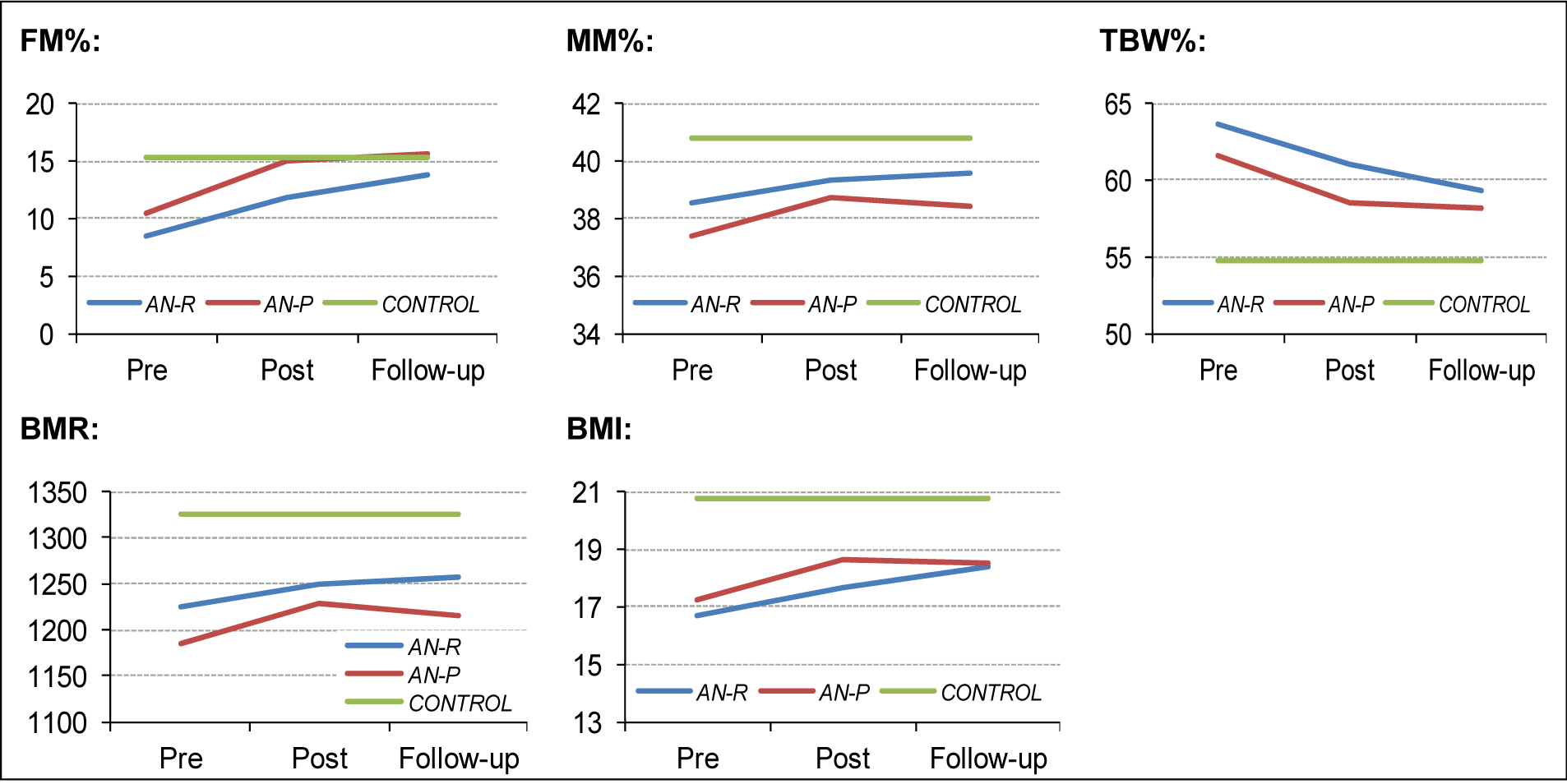

Supplement: S1 Fig — (TIF) [file pone.0143012.s001.tif]
